# A case of difficult‐to‐diagnose non‐invasive papillary squamous cell carcinoma of the uterine cervix infected with human papilloma virus 6: A diagnostic pitfall

**DOI:** 10.1002/ccr3.4905

**Published:** 2021-10-04

**Authors:** Kenbun Sone, Futaba Inoue, Ayumi Taguchi, Munetoshi Hinata, Masako Ikemura, Yuichiro Miyamoto, Tanikawa Michihiro, Tamami Ohno, Takayuki Iriyama, Mayuyo Mori‐Uchino, Tetsushi Tsuruga, Misako Mishima, Yutaka Osuga

**Affiliations:** ^1^ Department of Obstetrics and Gynecology Graduate School of Medicine The University of Tokyo Tokyo Japan; ^2^ Department of pathology Graduate School of Medicine The University of Tokyo Tokyo Japan; ^3^ Kawakita General Hospital Tokyo Japan

**Keywords:** condyloma acuminatum, human papillomavirus 6, papillary squamous cell carcinoma, papillomavirus infections

## Abstract

We encountered HPV6‐positive cervical papillary squamous cancer (PSCC) that was difficult to diagnose. The case was initially diagnosed and treated for condyloma. To the best of our knowledge, this is the first report of HPV6 infection in PSCC.

## Graphical abstract

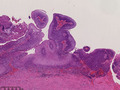

## 1 INTRODUCTION

We encountered a case in which a recurrent condyloma‐like papillary tumor was repeatedly treated as condyloma. Subsequently, the patient was re‐diagnosed and treated for non‐invasive cervical papillary squamous cancer (PSCC) with HPV6 infection. To the best of our knowledge, this is the first report of HPV6 infection in PSCC. Papillary squamous cell carcinoma (PSCC) of the uterine cervix is an extremely rare histological type of cervical cancer, which accounts for approximately 1.6% of all cervical cancers.^1^ PSCC cannot be easily diagnosed by colposcopy, and it is difficult to determine the degree of invasion.^1^ We diagnosed a patient with condyloma and treated her with for condyloma several times, but the patient relapsed repeatedly. She was re‐diagnosed as having PSCC and underwent surgery. We present this case report and discuss the diagnosis of PSCC and its pitfalls. In addition, we discovered that the PSCC was infected with HPV6, which has not been previously reported.

## 2 CASE PRESENTATION

A 47‐year‐old woman with one pregnancy and one parity, and no relevant medical, family, or menstruation history, presented to our hospital. The patient had been followed up from CIN 1 to 2 at a previous hospital for 8 years. A high‐risk HPV test was negative twice. Subsequently, a papillary mass was found in the cervix, and histological examination of the cervix suggested CIN 3 or condyloma. Conization was performed at another hospital, and the diagnosis was cervical condyloma with a positive margin.

Subsequently, the patient returned to the previous hospital and was followed up. However, 3 months later, there was recurrence of papillary tumor in the cervix; a biopsy was performed, which led to the diagnosis of condyloma. Cervical laser ablation was performed to treat the tumor. Two months later, the cervical tumor relapsed. Although laser vaporization was performed, the tumor relapsed again after 1 month. Cryotherapy was performed to treat the relapsed tumor, but the cervical tumor relapsed. Since the tumor recurred repeatedly in a short time, pathohistological examination was performed by a pathologist at another hospital and the possibility of PSCC was indicated. The patient was then referred to our hospital for examination.

**FIGURE 1:**
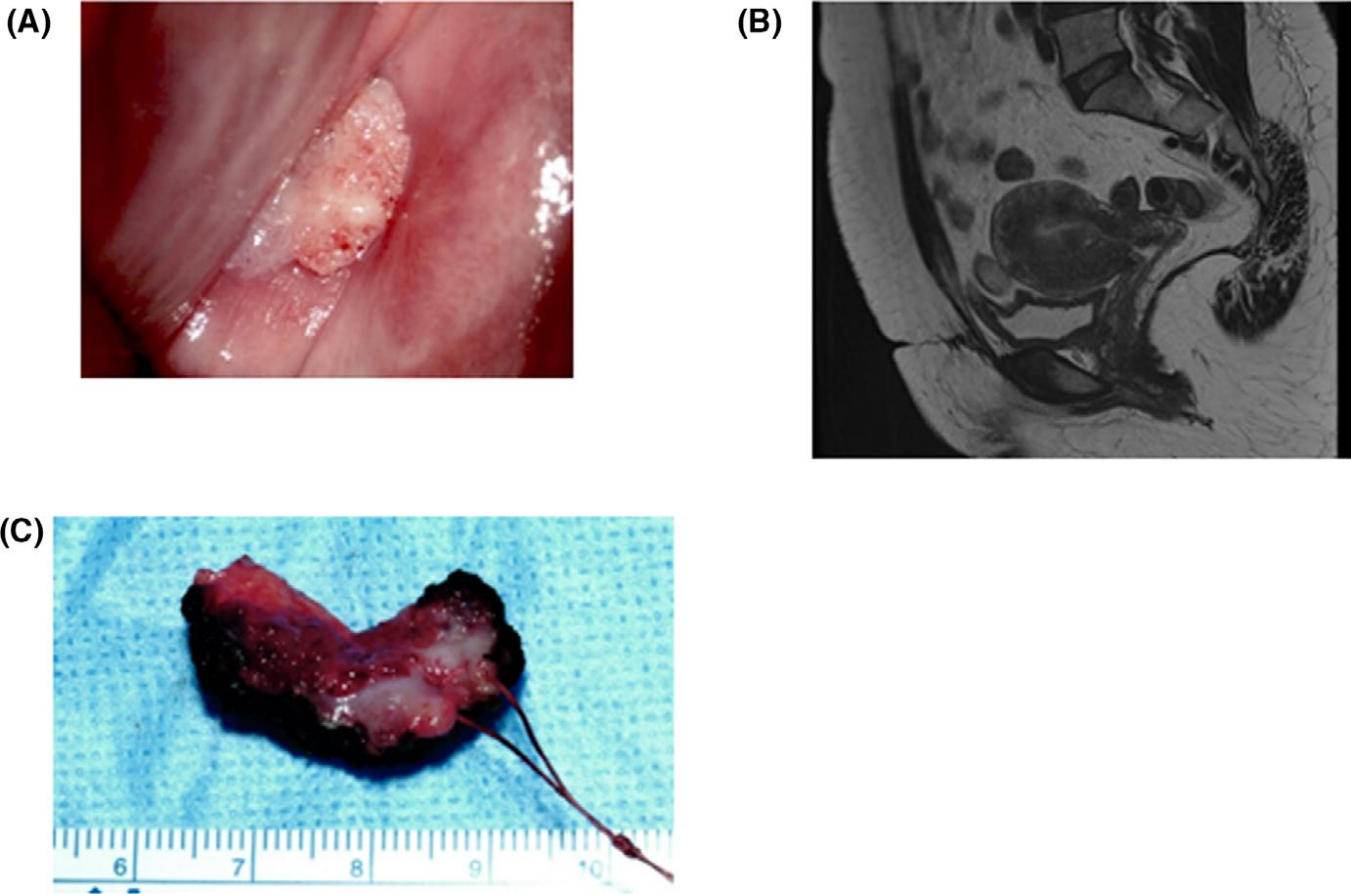
(A) Colposcopic findings. A papillary mass approximately 1.5 cm in diameter. (B) MRI T2‐weighted sagittal images. There is no obvious cervical mass. (C) Cervical conization specimen also showed a papillary mass in the cervix

**FIGURE 2:**
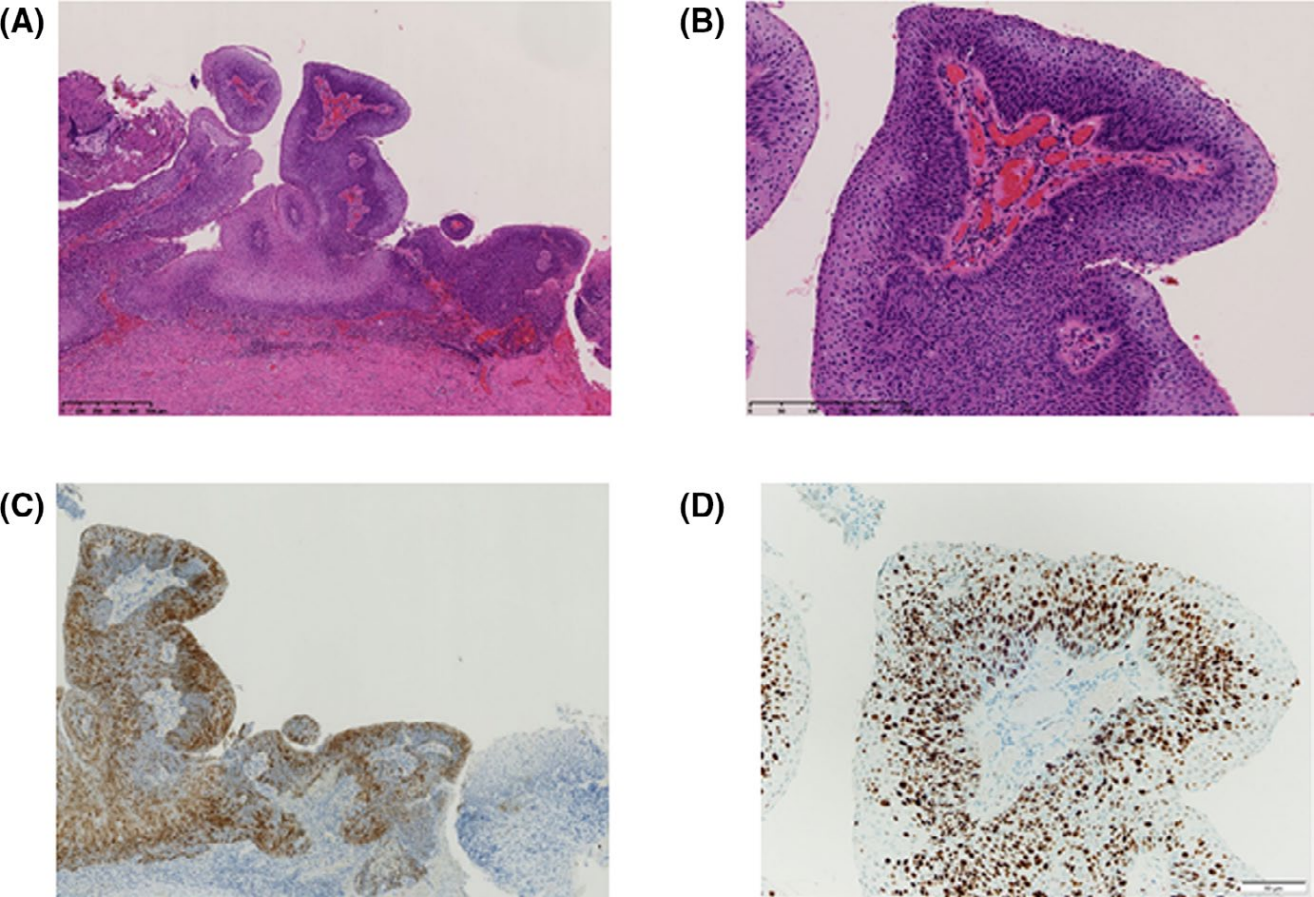
(A, B) Pathologic images of cervical conization specimens (HE staining). Full‐thickness dysplastic cells were observed in a papillary architecture with fibrovascular cores. No invasion was observed in this specimen. (A) Weak magnification. (B) Strong magnification. (C) Immunohistochemistry of cervical conization specimens: p16 was strongly positive. (D) Immunohistochemistry of cervical conization specimens: Ki67 was strongly positive

Colposcopy revealed a condyloma‐like papillary tumor approximately 1.5 cm in diameter (Figure 1A). No abnormalities were observed in other internal examination findings. Transvaginal ultrasonography revealed no abnormal findings in the uterus or ovaries. The results of a cervical biopsy under colposcopy were atypical epithelium, which was difficult to diagnose by biopsy alone. There were no apparent abnormalities in the MRI findings (Figure 1B), CT findings, or tumor markers. Second cervical conization was performed to confirm the diagnosis (Figure 1C), and the patient was diagnosed with non‐invasive PSCC (Figure 2A, B). Immunohistochemistry analysis showed that p16 and Ki67 were strongly positive in the surgical samples (Figure 2C, D). A cervical test for high‐risk HPV was negative in our hospital. Laparoscopic total hysterectomy and bilateral salpingo‐oophorectomy were performed. The patient was discharged with a good postoperative course and was followed up as an outpatient. After surgery for PSCC, DNA extracted from cervical tissue was subjected to HPV genotyping by Multiplex PCR, a rapid and sensitive PCR‐based HPV genotyping assay that can amplify 16 HPV genotypes (Types 16, 58, 52, 51, 56, 31, 18, 39, 66, 59, 6, 33, 30, 35, 45, and 11) in a single reaction. Only HPV 6 was detected by Multiplex PCR. (Figure S1).

## 3 DISCUSSION

We encountered a case in which a condyloma‐like papillary tumor was repeatedly treated as condyloma; however, there was repeated recurrence. Therefore, the patient was re‐diagnosed and treated for non‐invasive cervical cancer with HPV 6 infection. In 1986, Randall reported nine cases of PSCC for the first time worldwide. The characteristic pathological finding of PSCC is full‐thickness dysplastic cells in a papillary architecture with fibrovascular cores and an invasive component that is usually deep to the papillary excrescences.^2^ Colposcopy findings of PSCC are characterized by the development of a condyloma‐like mass with exophytic growth.^1^ However, they clearly distinguished PSCC from squamous papilloma, condyloma, and verrucous carcinoma.^2^ Compared to conventional SCC, PSCC is characterized by slowly progressing lesions. In addition, the possibility that PSCC is different from traditional SCC, in terms of the high frequency of late metastasis and recurrence, is also indicated.^3^ For instance, Randall reported that two cases of PSCC recurred over more than 7 years (87 months and 106 months) after resection, and Koenig reported vaginal recurrence of PSCC 12 years after the initial diagnosis.^2^, ^3^ Because PSCC is a rare histological type, the treatment strategy is the same as that for conventional SCC.^1^, ^3^

In this case, treatment for condyloma was performed several times for non‐invasive PSCC. However, despite inadequate treatment for non‐invasive PSCC, the tumor did not invade or spread, indicating slow progression of PSCC, as previously reported.^1^ It is also known that PSCC is difficult to diagnose preoperatively. Nagura et al. reported 28 cases of PSCC diagnosed by colposcopic biopsy, of which 12 (43%) were true PSCC; the other cases were non‐keratinized or microinvasive SCC.^1^ They also reported that PSCC is difficult to diagnose via biopsy specimens because of the degree of stromal invasion. If MRI shows stromal invasion of ≥3 mm, radical hysterectomy or radical trachelectomy is considered. It has been reported that conization or simple hysterectomy is considered if stromal invasion of ≤3 mm is suspected on MRI.^1^

It is also necessary to pay attention to the difficulty in diagnosis and pitfalls, and to consider PSCC when an intractable condyloma‐like mass is observed. Additionally, it is necessary to consider a re‐diagnosis of the pathology if PSCC is suspected. It has been reported that the positive rate of high‐risk HPV in PSCC is lower than that in cases of conventional SCC (50% vs. >95%). High‐risk HPV positivity in PSCC is often associated with HPV type 16.^4^, ^5^ Immunochemistry data from previous reports showed that the expression of Ki67, p63, and p16 is higher in PSCC than in condyloma.^6^, ^7^ A strong positive result for p16 was also observed in our case. E‐cadherin and N‐cadherin have been reported to be positive in SCC by immunostaining. Jang et al. reported that expression of E‐cadherin was negatively correlated with the prognosis of SCC.^8^ In contrast, Chandolia et al. reported that N‐cadherin was highly expressed in oral SCC compared with normal tissues.^9^

We performed high‐risk HPV screening tests several times before surgery, but they were negative; however, HPV 6 was identified by HPV DNA genotyping of tumor samples after surgery. Only HPV 6 was specifically detected in cryosurgery specimens using the Multiplex PCR method. The Multiplex PCR method allows for rapid, highly sensitive, and simultaneous measurement of 16 HPV genotypes (Types 16, 58, 52, 51, 56, 31, 18, 39, 66, 59, 6, 33, 30, 35, 45, and 11) in a single step. The primer set is designed to avoid false negatives. The results of the Multiplex PCR method and preoperative high‐risk HPV genotyping suggest that the patient was infected with HPV 6 rather than high‐risk HPV.

To the best of our knowledge, this is the first report of HPV 6 infection in PSCC. Although HPV 6 is not a high‐risk HPV, HPV 6 infection was previously observed in CIN 2/3,^10^ and integration of HPV 6 integration was reported as causing malignant transformation in a case of laryngeal cancer.^11^ It is considered that HPV 6 infection can cause non‐invasive PSCC based on the above report and the slow progress in this case. Although the false‐negative and false positive rate of the Multiplex PCR method is low, it is not zero. The possibility of high‐risk HPV‐dependent or HPV‐independent carcinogenesis must also be considered. Therefore it is necessary to accumulate similar cases in the future. From the above, it is possible that the mechanisms of generation and progress of PSCC are different from those of conventional SCC. However, further investigation is necessary in the future.

## CONFLICT OF INTEREST

We have no conflicts of interest to declare regarding this case.

## AUTHOR CONTRIBUTIONS

KS, FI, AT, YM, and TO analyzed and interpreted data; KS, MH, and MI prepared the manuscript and figures; MT, TI, MMU, and TT reviewed the manuscript; and KS, MM, and YO revised the manuscript for important intellectual content. All authors have read and approved the final version of this manuscript.

## ETHICAL APPROVAL

The study design was approved by the ethics committee of the University of Tokyo.

## CONSENT

The patient provided written informed consent for the publication of any associated data and accompanying images.

## Supporting information

Fig S1

## Data Availability

Not applicable.
