# Supplementary figures and images for: A case of difficult‐to‐diagnose non‐invasive papillary squamous cell carcinoma of the uterine cervix infected with human papilloma virus 6: A diagnostic pitfall
Source: Clin Case Rep. 2021 Oct 4;9(10):e04905. doi: 10.1002/ccr3.4905 (PMC8489388; doi:10.1002/ccr3.4905)

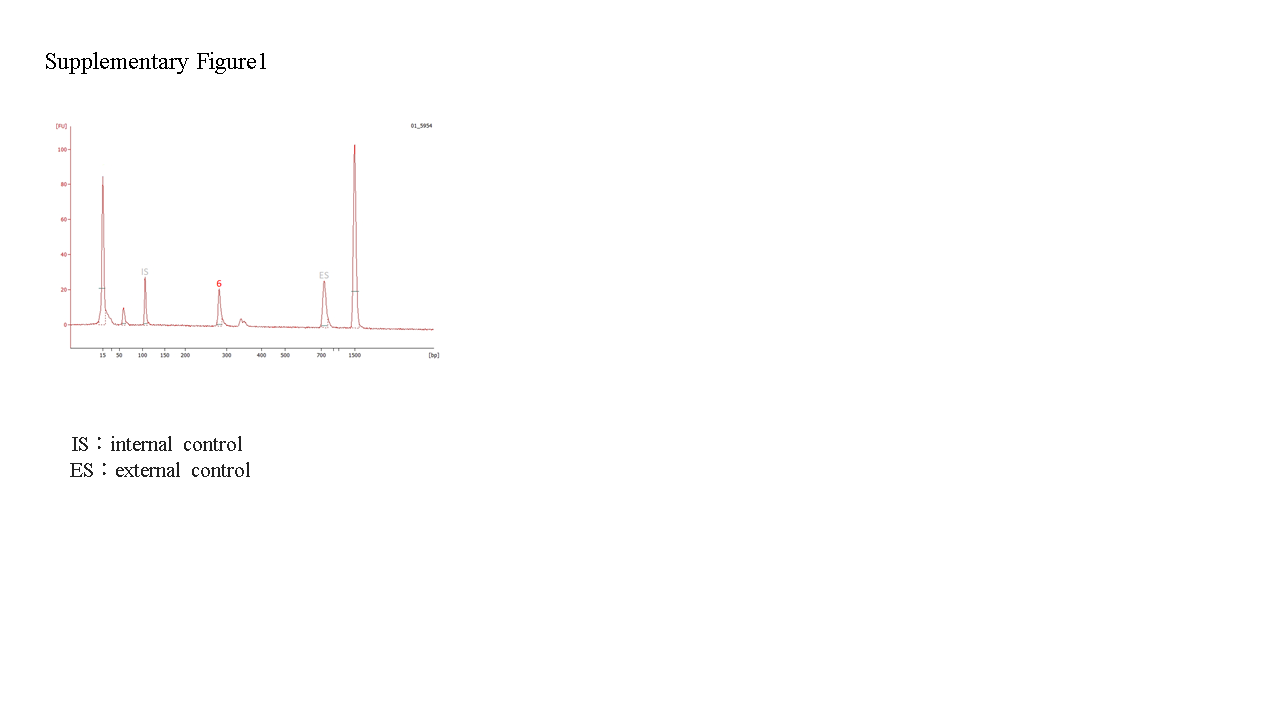

Supplement: Supplementary file 1 — Fig S1 [file CCR3-9-e04905-s001.TIF]
